# Assessment of Selected Parameters of Liver Fibrosis and Inflammation in Patients with Diagnosed Cystic Fibrosis

**DOI:** 10.1155/2020/5696185

**Published:** 2020-03-25

**Authors:** Sabina Więcek, Halina Woś, Andrzej Pogorzelski, Bożena Kordys-Darmolinska, Henryk Mazurek, Urszula Grzybowska-Chlebowczyk

**Affiliations:** ^1^Department of Pediatrics, Medical University of Silesia, Katowice, Poland; ^2^Upper Silesian Child Health Centre, Katowice, Poland; ^3^University of Bielsko-Biała, Poland; ^4^Department of Pneumonology and Cystic Fibrosis, Institute of Tuberculosis and Lung Disorders, Rabka-Zdrój, Poland

## Abstract

Changes in the liver and bile ducts observed in patients diagnosed with cystic fibrosis result from inflammatory processes as well as fibrosis, remodeling, apoptosis, and cholestasis. As a consequence, portal hypertension, cirrhosis, and hepatic failure may develop. So far, the complexity of these processes has not been elucidated. *Study Objectives*. The aim of the study was to evaluate the selected parameters of hepatitis and fibrosis (Fibrotest, Actitest, and APRI) in patients diagnosed with cystic fibrosis. *Material and Methods*. The study included 79 patients with cystic fibrosis, aged 1 to 20 years (mean age 9.8 years), 49 girls (62%) and 30 boys (38%). The analysis involved the following: age, sex, clinical manifestations, laboratory tests evaluating pancreas function, parameters of liver damage, and cholestasis. Fibrotest, Actitest, and APRI were performed in all subjects. *Results*. Elevated parameters of hepatic cell damage (hypertransaminasemia) were found in 31/79 (39.2%) patients, while abnormal cholestasis parameters in 21/79 (26.6%). The abnormal results of Fibrotest were reported in 15% of patients (12/79), while of Actitest in 10% (8/79). In contrast, elevated APRI values were found in only 7.6% (6/79) of subjects. There was a statistically significant correlation between APRI and age (higher values were observed in younger children) and between Fibrotest and Actitest and pancreatic insufficiency (higher values were found in subjects without this abnormality). Moreover, Fibrotest values were significantly higher in girls. There was no correlation between Fibrotest, Actitest, and APRI values and the type of mutation. *Conclusion*. It appears that Fibrotest may be used as an early marker of liver fibrosis in patients with cystic fibrosis. Increased APRI values were only found in subjects with advanced hepatic lesions, most often in the form of portal hypertension.

## 1. Introduction

Although hepatic changes occur only in 5-30% of patients diagnosed with cystic fibrosis (CF), they increase mortality and deteriorate the quality of life. Liver diseases are the most common extrapulmonary causes of death in patients with cystic fibrosis. They occur most often in the first decade of life [1–4]. Cirrhosis of the liver is detected in approximately 10% of children (<18 years) with cystic fibrosis, compared to only 2% of adults with this disease. The mean age of the onset of liver cirrhosis is approximately 10 years [4–7]. Liver damage observed in the course of cystic fibrosis involves a group of complicated processes of fibrogenesis, inflammation, remodeling, apoptosis, and cholestasis. The complexity of processes which take place in the liver and bile ducts in the course of this disease has not been explained. The role of pathophysiological lesions of bile acids and genetic and immunological factors is highlighted in the etiopathogenesis of hepatic lesions in cystic fibrosis [1, 2, 5]. Abnormal expression of cystic fibrosis transmembrane conductance regulator (CFTR) protein on the apical membranes of cholangiocytes within the epithelium of the bile ducts and the epithelium of the gallbladder leads to changes to the composition of bile, abnormalities to its transport, and the retention of toxic bile acids (taurine), which induces chemokines responsible for the processes of inflammation and fibrosis [2, 5, 6]. The most common lesions to the liver in the course of CF include focal liver fibrosis (72%), focal biliary cirrhosis (20-30%), multilobular biliary cirrhosis (5-15%), portal hypertension (2-5%), and/or cholelithiasis (14-24%). [1, 3, 6, 7] In most patients with cystic fibrosis, there are no symptoms of hepatic complications. In those with a more advanced stage of the condition, sometimes itchiness and jaundice are observed. Incidentally, diagnosed hepatomegaly is usually the first symptom. Also, the symptoms of portal hypertension—bleeding from the esophageal varices and/or splenomegaly—can be considered the first symptoms. Hepatic steatosis may be incidentally diagnosed in neonates during routine abdominal ultrasound [2, 4, 6–9].

Less invasive techniques developed for adults have been applied in pediatric patients with chronic diseases [10–12]. Laboratory tests assessing the processes of hepatic fibrosis in cystic fibrosis include simple measurement of hepatic parameters and complex index Fibrotest (involving alpha2-macroglobulin, A1 apolipoprotein, and haptoglobin), procollagen III aminopeptides, collagen I, collagen IV, laminin, hyaluronic acid, cytokines, and chemokines relating to fibrosis and cytokeratin 18. Most of the tests are only performed in a handful of laboratories. AST to Platelet Ratio Index (APRI) and Fibrotest, both used in the assessment of the processes of hepatic fibrosis, seem to be more available. Actitest, which additionally indicates the activity of ALT, may also prove useful [13–20].

### 1.1. The Aim of Study

The aim of the study was to assess and compare selected parameters of liver fibrosis and inflammation (Fibrotest, Actitest, and APRI) in patients with diagnosed cystic fibrosis.

## 2. Materials and Methods

We examined 79 patients with diagnosed cystic fibrosis, aged up to 1 year to 20 years (the average age of 9.8 years), 49 girls (62%) and 30 boys (38%), diagnosed and treated in the Department of Pediatrics of the Medical University of Silesia in Katowice and the Department of Pneumonology and Cystic Fibrosis of the Institute of Tuberculosis and Lung Disorders in Rabka-Zdrój.

### 2.1. Inclusion Criteria

The inclusion criteria involved the diagnosis of CF confirmed by genetic testing and aged up to 1 year to 20 years. Each patient or their legal guardian had signed an informed consent to participate in the study.

### 2.2. Exclusion Criteria

Patients aged less than 1 year, as well as those with acute infection or who received antibiotics for any cause during previous 4 weeks, were excluded.

The analysis included age, sex, clinical symptoms, laboratory tests for exocrine and endocrine pancreatic efficiency (elastase activity and acid steatocrit index in the stool, glucose concentration in the blood), laboratory parameters of liver damage, and cholestasis (activity of alanine and aspartate aminotransferases, alkaline phosphatase, gamma-glutamyltranspeptidase, concentration of albumin, bilirubin, alpha-macroglobulin, A1 apolipoprotein, and haptoglobin).

In diagnosing malnutrition, the BMI index was based, taking into account age, sex, and population. In addition, laboratory results (total protein, serum albumin) were included.

Fibrotest was calculated from the logarithmic equation taking the following parameters into consideration: alpha2-macroglobulin, A1 apolipoprotein, haptoglobin, the concentration of bilirubin, the activity of GTP and of aspartate aminotransferase, age, and sex. The values of <0.25 (F0) were considered normal.

For the calculation of Actitest, the activity of alanine aminotransferase was also considered. The values of <0.25 (A0) were considered normal.

The concentration of alpha2-macroglobulin was assessed using the immunoturbidimetric method and the photometric system using reagents manufactured by DiAgam. Depending on the sex, the values considered normal were the concentrations of 1.5-3.5 g/l (M)/1.75-4.20 g/l (F).

The concentration of apolipoprotein A1 was assessed using the immunoturbidimetric method and the reagents manufactured by Roche/Hitachi cobas. Depending on the sex, the values considered normal were the concentrations of 1.04- 2.02 g/l (M)/1.08-2.25 g/l (F).

The concentration of haptoglobin 2 was determined using the immunoturbidimetric method with the reagents manufactured by Roche/Hitachi cobas. Values from 0.3 to 2.0 g/l were considered normal.

APRI was calculated using the formula of Wai et al. [21] [AST/upper limit of normal (ULN)/platelet count (expressed as platelets × 10^9^/l) × 100]. The APRI value was calculated in patients (on an empty stomach), following the period of at least eight hours fasting, with no indicators of an acute infection.

The ultrasonography examinations of the abdomen with Doppler option were performed in all patients with diagnosed cystic fibrosis.

The study protocol was approved by the Bioethics Committee of the Silesian Medical University in Katowice (KNW/0022/KB1/158/2014).

### 2.3. Statistical Analysis

The statistical analysis was based on the procedures set forth in the MedCalc 14.8.1 licensed software (MedCalc Software bvba, Ostend, Belgium). The quantitative variables were given as average and a standard deviation (variables of normal distribution) or medians and interquartile range (skewed distribution). The distribution of the variables had been verified with D'Agostino-Pearson's test. The qualitative variables were presented as absolute values and percentage. Differences between the groups for quantitative variables were calculated using *t*-Student or ANOVA tests (variables of normal distribution) or *U* Mann-Whitney or Kruskal-Wallis (skewed distribution). The Chi-square test or Fisher's exact test were used for qualitative variables. The correlations were interpreted using Spearman's rank correlation. The results of simple analyses were verified using multiple variables' analysis in the multiple linear regression. Variables of *p* < 0.1 in simple analyses were added to the model. The criterium of statistical significance was agreed at *p* < 0.05.

## 3. Results

Clinical symptoms reported by examined patients with diagnosed cystic fibrosis dominated pancreatic failure (87.34%), respiratory tract symptoms (81.0%), and malnutrition (53.2%). In 11.4%, we observed electrolyte disturbance (salt lost syndrome in the history), and in 5.1%, history of *meconium ileus* was positive. Shwachman-Kulczycki scale was 50.0–110.0 point (mean 75.56). Disturbances of the carbohydrate metabolism were observed in one of the patients. None of the patients with diagnosed cystic fibrosis in the examined group has innovative therapies with lumacaftor in combination with ivacaftor.

### 3.1. Genetic Examinations

The mutation delF508/delF508 was observed in 41/79 patients (51.9%). The mutation of delF508/another was less frequent in 32/79 (40.5%) (Table 1).

Cystic fibrosis liver disease (CFLD) based on the Debray et al.'s criteria (hepatomegaly/abnormalities in liver function test/ultrasonography abnormalities) was diagnosed in 19/79 (24.1%) patients with cystic fibrosis.

The above-mentioned parameters of damage to the liver were observed in over 1/3 of the patients, and they manifested themselves mostly as elevated activity of aspartate aminotransferase (31/79 (39.2%)) and/or elevated activity of alanine aminotransferase (19/79 (24.1%)). Elevated parameters of cholestasis were observed in over 1/4 of the patients—mainly as elevated activity of gamma-glutamyltranspeptidase (21/79 (26.6%)) (Table 2).

Abnormal values in the Fibrotest were reported in 15% (12/79) of patients with diagnosed cystic fibrosis and 10% in Actitest (8/79). Abnormal APRI was observed in only 7.6% (6/79). Correlations between the tests are presented in Table 3.

Abnormalities in the abdominal ultrasound (most frequently in the form of steatosis, hepatomegaly, and/or portal hypertension) were demonstrated in 32/79 patients (enlarged liver 19/79 (24.0%), steatosis of liver 20/79 (25.31%), and cholelithiasis -3/79 (3.8%)).

Abnormalities of the portal system evidenced in the Doppler examination were concluded in 8/79 (10.12%) of the patients in the form of portal hypertension, changes to the vascular flow, and splenomegaly.

Statistically significant discrepancies were reported between the age and the APRI, the highest values in the youngest children. No correlations were observed between the values of Fibrotest, Actitest, and APRI—and the type of CFTR gene (Table 4(a)). The Fibrotest values for girls were statistically significantly higher 0.17 vs. 0.1 (*p* < 0.001).

All analyzed patients with abnormal APRI had CFLD diagnosed based on Debray criteria (6/19 (31.57%)). The average APRI values were also statistically significantly higher in this group of patients (0.52 vs. 0.32).

The mean Fibrotest values were statistically significantly higher in the CFLD group (0.22 vs. 0.10). However, elevated Fibrotest values were also observed in 1 patient without diagnosed CFLD.

There was no correlation between Actitest values in patients with or without CFLD (0.12 vs. 0.10).

Having analyzed the clinical symptoms, statistically significant discrepancies were concluded between the values of Fibrotest and Actitest and pancreatic insufficiency—higher values were reported in patients without pancreatic abnormalities. Higher values of Actitest were also concluded in malnourished patients (*p* = 0.047). Statistically significantly higher values of APRI were reported in patients with the normal functioning of the respiratory system (*p* = 0.005) (Table 4(b)).

## 4. Discussion

The role of the pathophysiological changes to bile acids and of genetic and immunological factors is of importance in the etiopathogenesis of hepatic lesions in the course of cystic fibrosis [1, 3, 5, 22]. The frequency of the occurrence of lesions to the liver and bile ducts in patients with cystic fibrosis is estimated at 5-30% [2–5, 23–25].

The subject literature contains numerous reports on the occurrence of cystic fibrosis liver disease (CFLD) among the adult population but not that many concerning pediatric patients. Chryssostalis et al. reported the presence of lesions in the liver in 90 out of 285 adult patients (32%) over the age of 18 (the average age of 34.5 yo) with diagnosed cystic fibrosis and liver cirrhosis in 23 (8%). The factors which he considered prognostically disadvantageous in the progression of hepatic lesions include the history of meconium ileus, colonisation with Burkholderia cepacia, and intravenous antibiotic therapy [26]. In turn, Sadler et al. reported the presence of CFLD lesions in only 14% of patients with cystic fibrosis. They were younger patients, aged from 22 to 37 with an average age of 27 years [27]. As for our patients, we observed hepatic lesions in over 30%, mainly in the form of elevated activity of aminotransferases to the liver and/or cholestasis. Cystic fibrosis liver disease (CFLD) based on Debray et al.'s criteria was diagnosed in 19/79 (24.1%) of our patients with cystic fibrosis. Stonebraker et al., having analyzed a group of patients aged 2 to 52 (the average of 18), reported hepatic lesions in over 40% of patients with CF, 16% of whom (90/561) required liver transplant [28]. Similarly, Staufer et al., in her study of CF patients aged 23 to 43 (the average of 30.3), concluded the presence of CFLD-type lesions in 30% of the patients. The second decade of life is the time when changes to the liver and bile ducts in the course of cystic fibrosis are most commonly observed. Factors leading to their occurrence have been investigated for years [29]. The most similar group of examined patients in respect of age was a group examined by Polineni et al. Polineni et al. in her study of 156 patients with CFLD aged 1 to 28 concluded 12 years of age as the average age when lesions in the liver and/or bile ducts are observed [30]. We observed the above-mentioned changes in our 2-to-6-year-old patients, but the studied group involved those up to the age of 20 (the average age of 9.8). Bhardwaj et al. in his study of 263 patients with cystic fibrosis, both adults and children observed CFLD-like lesions in 65% of patients, with high incidence among those under the age of 18 (84% vs. 16%). He concluded that the risk of developing hepatic lesions lessens with age, by about 10% every year, and that such abnormalities very rarely manifest themselves below the age of 18 [31]. Stonebraker et al. observed the occurrence of hepatic lesions most often around the age of 10, and the average age of liver transplant due to CFLD was 13.9 [28]. On the contrary, Karlas et al. and Ayoub et al. did not observe any correlation between the age and the occurrence of CFLD-type lesions [32, 33]. The literature on the subject includes data reporting a frequent occurrence of CFLD among men. The data was confirmed by Stonebraker et al. and Keyte et al. [28, 34] as well in our study. In terms of our patients, girls were affected by the hepatic lesions more frequently. However, Karlas et al. and Ayoub et al. did not prove a link between CFLD and the sex [32, 33].

Genetic background undoubtedly plays a role in the occurrence of hepatic lesions in the course of cystic fibrosis. As Rowland et al. pointed out, “severe mutations” of CFTR gene (i.e., delta F508, G524X, N1303K, CFTRdel21kB, and 1811+1G-> C) are observed in CF patients with cystic fibrosis [24]. Keyte et al., Rowntree and Harris, and Flass and Narkewicz observed that there was a link between the occurrence of “severe mutations” (class I, II, and III) and higher mortality, pancreatic insufficiency, history of meconium ileus, and abnormalities in the liver [34–36]. We did not conclude a link between hepatic lesions and type of CFTR mutation, possibly because a severe mutation of deltaF508/deltaF508 was reported in over 50%. Another limitation of our study was a relatively low number of subjects. Ledder et al., Indika et al., and Stonebraker et al. did not report any correlation between phenotype and genotype in patients with cystic fibrosis and accompanying hepatic lesions. What they did report were severe mutations of CFTR gene [28, 37, 38].

Ayoub et al. did not show a link between hepatic lesions in the course of cystic fibrosis and the clinical picture (pancreatic insufficiency and malnutrition) [33]. Similarly, there were not many cases of our CF patients with advanced pancreatic insufficiency and abnormalities in the respiratory system and the occurrence of hepatic lesions. In his study of patients with cystic fibrosis in need of lung transplant, Karlas et al. did not confirm a link between the occurrence of hepatic lesions and the level of nutrition, the duration of the condition, and the age at the time of diagnosis [32]. Hepatic lesions in the form of CFLD were only observed in 12% of the patients. Stonebraker et al., however, observed pancreatic exocrine insufficiency in almost all of his patients with CFLD [28].

The role of malnutrition is often highlighted in the pathogenesis of hepatic lesions. Patients with cystic fibrosis have significantly lower levels of linolenic, docosahexaenoic, and docosapentaenoic acids. Insufficient amounts of antioxidants and vitamins and frequently performed parenteral nutrition play an additional role [4, 8, 24]. Over half of our patients were underweight (53%). Early studies by Rowland et al., Debray et al., and Karlas et al. of CFLD associated the finding of hepatic steatosis with severe malnutrition while others have associated it with essential fatty acid deficiency [4, 8, 24, 32].

There is, however, a growing number of overweight patients with cystic fibrosis. Ayoub et al. observed fatty liver in over 14% of CF patients, with the body mass index exceeding 25 in most cases and no symptoms of portal hypertension present in endoscopic and imaging tests. It appears that this may be related to the ever more effective pharmacological and dietary treatment and growing obesity leading to NASH-type (nonalcoholic steatohepatitis) abnormalities [33]. In our patients, the symptoms of fatty liver were observed during abdominal ultrasound in 20/79 (25.3%) with only 2 patients classified as obese in the physical examination. The symptoms of damage to the liver and cholestasis in the course of cystic fibrosis are not characterized and often present only at a very advanced stage when diagnosed. This makes the need for introducing minimally invasive tests which would allow for an early detection of early lesions in the liver even greater [39–41]. Early detection of fibrotic lesions seems to be particularly difficult. Among laboratory tests assessing the processes of liver fibrosis in the course of cystic fibrosis, the most widely available is APRI and Fibrotest, with Actitest as a marker of inflammatory processes [14, 39]. APRI is an indirect biochemical marker of hepatic fibrosis. There are little data available on the use of APRI in pediatric patients mainly with biliary atresia, chronic hepatitis B or C, NAFLD (nonalcoholic fatty liver disease), and intestinal failure [40–46]. Individual studies have analyzed the suitability of the APRI in adult patients with cystic fibrosis, and it is mainly the first and second decade of life when hepatic lesions occur. The latest studies have shown that APRI < 0.3 (0.5) rules out liver fibrosis and at a cut-off of 1.22 could predict macroscopic cirrhosis with a sensitivity of 75% and specificity of 84% [17]. In our patients, the APRI values ranged from 0.12 to 3.4, and only in 6/79 of patients (7.6%), the values exceeded 1.0. In all the patients, the hepatic lesions were advanced, mainly in the form of liver cirrhosis with accompanying portal hypertension. CFLD was diagnosed in all these patients with elevated APRI based on the Debray criteria. Many studies highlight the role of Fibrotest and Actitest as minimally invasive tests assessing liver fibrosis and inflammation [46–50]. Elevated values in Fibrotest were observed in 15.2% of our patients (12/79) and those in Actitest in 10.12% (8/79). The mean Fibrotest values were statistically significantly higher in the CFLD group (0.22 vs. 0.10). However, elevated Fibrotest values were also observed in 1 patient without diagnosed CFLD.

Studies which describe the suitability of Fibrotest in the early detection of hepatic lesions in the course of cystic fibrosis are scarce. Sadler et al., having analyzed 127 young adults with diagnosed cystic fibrosis (the average age of 27) using Fibrotest, APRI, and elastography, concluded the presence of hepatic lesions in 14% of patients, with Fibrotest showing the highest sensitivity to the early detection of early lesions [27]. Friedrich-Rust et al. compared the detection of hepatic fibrosis in CF patients using transient elastography (TE), acoustic radiation force impulse (ARFI), and Fibrotest—their sensitivity being 17%, 24%, and 16%, respectively [18]. The results of the tests were affected by the copresence of pancreatic insufficiency. In our patients, statistically significantly higher values of Fibrotest and Actitest were concluded in patients without accompanying pancreatic insufficiency. Such correlation was not observed with regard to APRI. In our studies, all patients with elevated values of APRI had also higher values of Fibrotest, but some of the patients with elevated values of Fibrotest had normal values of APRI. No abnormalities were observed in those patients in the abdominal ultrasound with Doppler of the portal system. It seems that elevated values of APRI are observed in patients with advanced hepatic lesions. Fibrotest may be used for the early detection of hepatic lesions. Leung et al., who compared the efficiency of APRI to the histopathological assessment of the liver in CF patients, concluded that APRI had a better sensitivity and specificity than Fibrotest in patients with advanced cirrhosis (sensitivity 95%, specificity 72%) [48].

## 5. Summary

It seems that Fibrotest can be an early marker of liver fibrosis in children with cystic fibrosis. Increased APRI was only observed in patients with advanced changes in the liver—portal hypertension.

It seems that Fibrotest should become a routine test performed in children with suspected CFLD.

## Figures and Tables

**Table 1:** Clinical picture of the examined patients.

	Values
Age (years)	9.8 (range: up to 1 year–20 years)
Sex female/male (*n*/*n*)	49/30
Mutation (*n* (%))	
delF508del/delF508del	41/79 (51.9%)
delF508del/other	32/79 (40.5%)
Other	6/79 (7.6%)
Shwachman-Kulczycki score (pts)	75.56 (range 50-110)
Clinical features (*n* (%))	
Pancreatic insufficiency	69/79 (87.34%)
Symptoms from the respiratory tract/recurrent respiratory tract infection	64/79 (81%)
Malnutrition (body mass index < 3pcn, taking into account age, sex, and population and laboratory test including albumin, total protein level)	42/79% (53.2%)
Liver dysfunction	31/79 (39.2%)
Salt loss syndrome	9/79 (11.4%)
History of meconium ileus/treated surgically	4/79 (5.1%)

**Table 2:** Parameters of liver function and fibrosis in examined patients.

Parameters	Range of activity	Mean activity	Number of patients with abnormality
Aspartate aminotransferase (AST)	10.0-151.0 (U/l)	44.025 (U/l)	**31/79 (39.2%)**
Gamma-glutamyltranspeptidase (GTP)	6-314 (U/l)	47.3 (U/l)	**21/79 (26.6%)**
Alanine aminotransferase (ALT)	9.0-142.0 (U/l)	34.57 (U/l)	**19/79 (24.1%)**
Alkaline phosphatase (AF)	111-697 (U/l)	343.9 (U/l)	**11/79 (13.9%)**
Bile acids	1.1-86 (*μ*mol/l)	15.6 (*μ*mol/l)	**8/79 (10.1%)**
Total bilirubin	1.6-28.4 (*μ*mol/l)	9.35 (*μ*mol/l)	**6/79 (7.6%)**
Alpha2-macroglobulin (g/l)	1.8-4.76	3.50	**28/79 (35.4%)**
Apolipoprotein A1 (g/l)	0.57- 2.03	1.36	**12/79 (15.2%)**
Haptoglobin (g/l)	0.03-3.54	1.25	**19/79 (24.05%)**

**Table 3:** Correlation between parameters of fibrosis an APRI in patients with cystic fibrosis.

Parameters	Range of activity	Mean activity	Number of patients with abnormality	Correlation with APRI
Fibrotest	0.02-0.55	0.138	**12/79 (15.2%)**	**p** < 0.05**(p** = 0.0065**)**
Actitest	0.01-0.65	0.10	**8/79 (10.12%)**	**p** < 0.05**(p** = 0.099**)**
APRI	0.12-3.40	0.458	**6/79 (7.6%)**	**—**

**Table:** (a) Values of APRI, Fibrotest, and Actitest depending on the clinical picture of patients with diagnosed cystic fibrosis

	Number	APRI range (mean)	Fibrotest	Actitest
Age				
<2 yo	13/79 (16.5%)	0.15-3.4 (mean: 0.33)	0.06-0.21 (mean: 0.13)	0.01-0.14 (mean: 0.092)
2-6 yo	29/79 (36.7%)	0.12-2.57 (mean:0.40)	0.100-0.55 (mean: 0.16)	0.07-0.65 (mean: 0.12)
>6 yo	37/79 (46.8%)	0.06-2.15 (mean:0.22)	0.02-0.165 (mean: 0.122)	0.04-0.100 (mean: 0.091)
		*p* = 0.008	*p* = 0.33	*p* = 0.18
Sex				
Female	49/79 (62%)	0.12-3.4 (mean: 0.33)	0.02-0.041 (mean: 0.17)	0.01-0.65 (mean:0.103)
Male	30/79 (38%)	0.15-2.15 (mean: 0.298)	0.05-0.55 (mean:0.1)	0.04-0.49 (mean: 0.105)
		*p* = 0.79	*p* = 0.0007	*p* = 0.17
*CFTR* mutation				
delF508del/delF508del	41/79 (51.9%)	0.12-3.4 (mean: 0.48)	0.02-0.16 (mean: 0.12)	0.047-0.65 (mean: 0.11)
delF508del/other	32/79 (40.5%)	0.12-2.15 (mean: 0.40)	0.09-0.55 (mean: 0.16)	0.01-0.11 (mean: 0.09)
Other	6/79 (7.6%)	0.21-0.68 (mean: 0.40)	0.11-0.13 (mean: 0.11)	0.06-0.10 (mean: 0.086)
		*p* = 0.44	*p* = 0.21	*p* = 0.96

**Table:** (b) Values of APRI, Fibrotest, and Actitest in relation to the clinical picture of patients with diagnosed cystic fibrosis

	Number	APRI range (mean)	Fibrotest	Actitest
CFLD (cystic fibrosis liver disease using Debray criteria)	Yes *N* = 19/79	0.15-3.4 (mean: 0.52)	0.04-0.55 (mean: 0.22)	0.07-0.65 (mean: 0.12)
	No *N* = 60/79	0.12-0.6 (mean: 0.32)	0.02-0.41 (mean: 0.10)	0.01-0.33 (mean: 0.10)
		*p* < 0.05	*p* < 0.05	*p* = 0.24

Pancreatic insufficiency	Yes *N* = 69/79	0.12-3.4 (mean: 0.46)	0.02-0.10 (mean: 0.076)	0.01-0.14 (mean: 0.098)
	No *N* = 10/79	0.12-0.87 (mean: 0.32)	0.06-0.55 (mean: 0.12)	0.07-0.65 (mean: 0.14)
		*p* = 0.21	*p* = 0.01	*p* = 0.013

Malnutrition	Yes *N* = 42/79	0.12-3.4 (mean: 0.54)	0.02-0.55 (mean: 0.14)	0.04-0.65 (mean: 0.12)
	No *N* = 37/79	0.12-0.59 (mean: 0.33)	0.07-0.18 (mean: 0.13)	0.01-0.100 (mean: 0.09)
		*p* = 0.27	*p* = 0.66	*p* = 0.047

Symptoms from respiratory tract/recurrent respiratory tract infection	Yes *N* = 64/79	0.12-3.4 (mean: 0.34)	0.02-0.170 (mean: 0.13)	0.01-0.65 (mean: 0.10)
	No *N* = 15/79	0.21-2.57 (mean: 0.87)	0.10-0.55 (mean: 0.16)	0.05-0.140 (mean: 0.11)
		*p* = 0.005	*p* = 0.11	*p* = 0.28

Salt loss syndrome	Yes *N* = 9/79	0.15-2.57 (mean: 0.66)	0.11-0.55 (mean: 0.17)	0.07-0.65 (mean: 0.133)
	No *N* = 70/79	0.12-3.4 (mean: 0.412)	0.02-0.17 (mean: 0.13)	0.01-0.10 (mean: 0.099)
		*p* = 0.40	*p* = 0.11	*p* = 0.096

## Data Availability

The statistical analysis and database used to support the findings of the study “Assessment of selected parameters of liver fibrosis and inflammation in patients with diagnosed cystic fibrosis” may be released upon application to the Medical University of Silesia, Department of Pediatrics, who can be contacted by the corresponding author.
